# Hepatic Adrenal Rest Tumors Mimicking Hepatocellular Carcinoma: A Case Report and Review of the Literature

**DOI:** 10.7759/cureus.31343

**Published:** 2022-11-10

**Authors:** Ahmad Al-Taee, Danielle Capenter, Robert Garrett, Mustafa Nazzal, Brent Tetri

**Affiliations:** 1 Internal Medicine, Saint Louis University Hospital, Saint Louis, USA; 2 Pathology, Saint Louis University School of Medicine, Saint Louis, USA; 3 Radiology, Saint Louis University Hospital, Saint Louis, USA; 4 Surgery, Saint Louis University Hospital, Saint Louis, USA; 5 Hepatology, Saint Louis University Hospital, Saint Louis, USA

**Keywords:** rest, hepatocellular, tumor, hepatic, adrenal

## Abstract

Adrenal rest tumors are rare collections of aberrantly located adrenocortical tissue. They are most commonly found in the kidneys, and hepatic involvement is rare with few published case reports. When located in the liver, imaging findings are frequently indistinguishable from hepatocellular carcinoma (HCC), but when resected, histologic examination shows adrenocortical tissue. Here, we present a patient with a history of nonalcoholic steatohepatitis with advanced fibrosis who was identified as having HCC by cross-sectional imaging but was found to have a hepatic adrenal rest tumor (HART) after resection. HARTs can share imaging characteristics with HCC, and this alternative diagnosis should be considered, especially for hepatic segment VII lesions.

## Introduction

Adrenal rest tumors are defined as collections of aberrantly located adrenal tissue [1,2]. The kidneys are the most common location followed by genital structures [3]. Hepatic involvement is rare and published literature is limited to a few case reports. Patients are usually asymptomatic unless tumors are large or functional [4,5]. Tumor markers such as alpha-fetoprotein are usually within normal limits [6]. Hepatic adrenal rest tumors (HARTs) share many imaging findings with hepatocellular carcinoma (HCC) and therefore a correct preoperative diagnosis is rare [6]. HARTs have a predilection for the posterosuperior segment of the right hepatic lobe (segment VII). Histologic examination is the gold standard for diagnosis and typically shows benign adrenal tissue although malignant transformation has been described [4,5]. This article was previously presented as a meeting abstract at the 2020 American College of Gastroenterology meeting.

## Case presentation

A 72-year-old woman was referred to our hepatology clinic at Saint Louis University Hospital for the management of newly diagnosed HCC. She had a history of nonalcoholic steatohepatitis with stage 3 fibrosis, obesity, gastroesophageal reflux disease, obstructive sleep apnea, and hypertension. There was no history of tobacco, alcohol, or illicit drug use. She had no gastrointestinal symptoms. Vital signs were within normal limits. BMI was 33.8 kg/m^2^. Physical examination revealed a soft abdomen without tenderness or hepatomegaly. No stigmata of chronic liver disease were noted.

**Figure 1:**
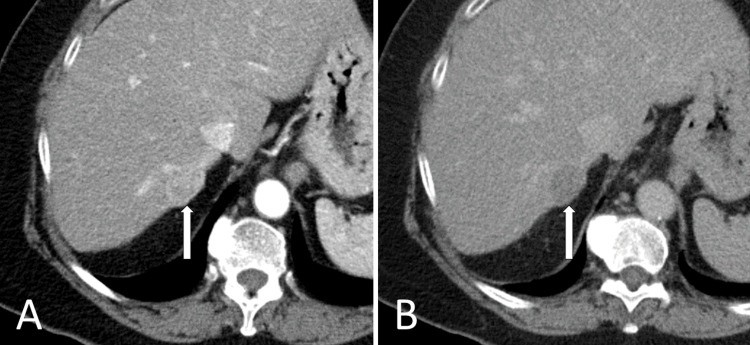
Liver protocol CT demonstrating LIRAD V lesion in segment 7 (A) Arterial phase, arterially enhancing lesion in segment 7; (B) Delayed phase demonstrates washout LI-RADS: Liver Imaging Reporting and Data System

Complete blood count and comprehensive metabolic panel were notable for elevated alanine aminotransferase of 113 U/L and aspartate aminotransferase of 65 U/L. Alkaline phosphatase, total bilirubin, hemoglobin, platelet count, and international normalized ratio (INR) were within reference ranges. Serum alpha-fetoprotein was 2.89 ng/ml. A right upper quadrant ultrasound, performed for screening for HCC revealed a 26-mm right hepatic lobe lesion. Dynamic MRI and CT imaging of the abdomen showed a 23-mm segment VII lesion that demonstrated arterial enhancement with washout on delayed phase, consistent with HCC as per the Liver Imaging Reporting and Data System (LI-RADS) criteria (Figure 1).

**Figure 2:**
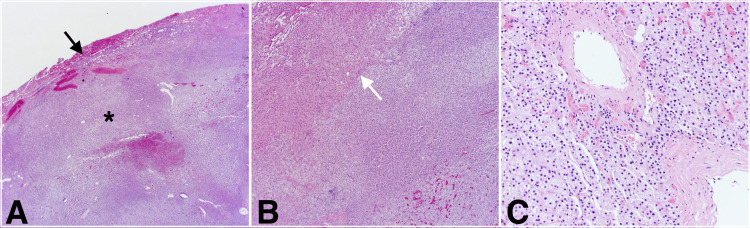
H&E stain slides (A) Low power magnification, adrenal rest tumor tissue (*) with scant peripheral hepatocellular tissue (black arrow); (B) Medium power magnification, interface (white arrow) between adrenocortical cells on the right and hepatocellular cells on the left; (C) High-power magnification, adrenocortical cells

**Figure 3:**
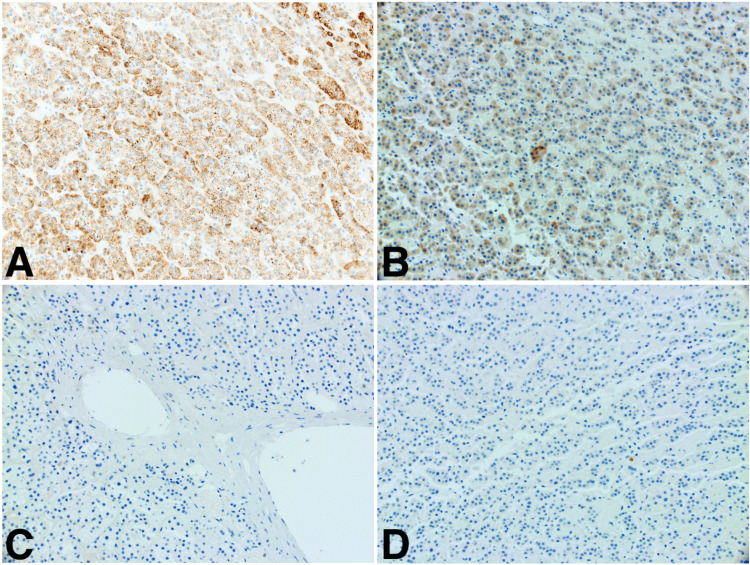
Immunohistochemical staining of surgical specimen confirming the adrenocortical origin (A) Positive for Mart-1/Melan; (B) Positive for inhibin; (C) Negative for hepatic-specific antigen; (D) Negative for PAX-8

She was evaluated for possible surgical resection with a transjugular liver biopsy showing steatohepatitis with stage 3 fibrosis and a hepatic vein pressure gradient measurement of 4 mmHg. As the patient was not a candidate for liver transplantation, given her age and morbid obesity, she elected to undergo partial hepatectomy of the segment VII lesion with ablation of the margins. The liver was noted to be nodular intraoperatively. Her postoperative course was unremarkable, and she was discharged home on day 3. Histologic examination of the resected lesion revealed benign adrenocortical tissue with clear margins (Figure 2). Immunostains were performed to confirm tissue identity, showing positive staining for Melan A, confirming adrenal origin, and negative staining for HSA and PAX-8, arguing against hepatocellular or renal origin respectively (Figure 3). Therefore, the diagnosis of HART was established.

## Discussion

Adrenal rest, also referred to as adrenal rest “tumor,” is a term used to describe ectopic adrenal tissue. The kidneys are most commonly involved followed by genital structures, and hepatic involvement is rare [3]. They are thought to arise during embryologic development as a fragment of the primitive adrenal gland breaks off and rests on another organ [6]. Patients are usually asymptomatic unless tumors are large, causing compression of the surrounding structures. In the liver, these tumors are rarely diagnosed preoperatively as imaging findings are frequently indistinguishable from HCC [7]. Histologic examination is the gold standard for diagnosis and typically shows benign adrenocortical tissue. Malignant transformation and hormone secretion have been reported in two cases [4,5]. As HCC, angiomyolipoma, and metastatic clear cell renal cell carcinoma are possible diagnoses, immunohistochemical staining can distinguish adrenal tissue from tissue of hepatic or renal origin [7].

We conducted a search of the English literature using the MEDLINE database for published cases of HARTs. We identified a total of 11 published case reports describing 11 patients [1,2,4-12]. Table 1 summarizes the published case reports. Age at diagnosis ranged from 21 to 67 years old with a median age at diagnosis of 58 years old. About 64% of cases were described in female patients. When tumor markers, such as alpha-fetoprotein, were checked, they were within normal limits. While the majority of cases were asymptomatic (82%), tumor secretion of glucocorticoids resulting in Cushing's syndrome has been described in two cases (18%) [4,5]. Our patient was reported to have hypertension that was difficult to control before surgery, but serum cortisol measurements were not obtained. Imaging findings included many of the features seen in HCC such as arterial enhancement and venous washout. Similar to our patient, the tumor was found in the posterosuperior segment of the right hepatic lobe (also referred to as hepatic segment VII) in almost all cases. The reasons for such anatomical predilection are not clear although this is anatomically adjacent to the right adrenal gland. Surgical resection was performed in all reported cases. Histologic examination revealed benign adrenocortical tissue in all cases except the two case reports describing functional tumors with malignant transformation.

**Table 1:** Summary of published case reports describing hepatic adrenal rest tumors

Authors and year of publication	Age and gender	Tumor size	Tumor location	Functional status	Malignant transformation	Country
Enjoji et al, 2017 [2]	67, female	17 mm	Segment 7	NO	NO	Japan
Sugiyama et al, 2015 [3]	50, female	30 mm	Segment 7	NO	NO	Japan
Wallace et al, 1981 [4]	23, female	150 mm	Right hepatic lobe	Yes	Yes	USA
Contreras et al, 1985 [5]	21, female	100 mm	Segment 7	Yes	Yes	Chile
Hyams et al, 1960 [6]	59, male	30 mm	Right hepatic lobe	No	No	USA
Arai et al, 2000 [7]	62, male	25 mm	Segment 7	No	No	Japan
Tajima et al, 2001 [8]	55, female	25 mm	Segment 7	No	No	Japan
Baba et al, 2008 [9]	67, female	15 mm	Segment 7	No	No	Japan
Shin et al, 2010 [10]	62, male	30 mm	Segment 7	No	No	Korea
Dalla Valle et al, 2014 [11]	58, male	25 mm	Segment 7	No	No	Italy
Soo et al, 2014 [12]	47, female	34 mm	Segment 7	No	No	Singapore

The finding of HART in the setting of chronic liver disease has been described in only one case report earlier [11]. Dalla Valle et al. reported HART in a 58-year-old man with a history of hepatitis B and human immunodeficiency virus co-infection. As HARTs share many of the radiologic features of HCC, they can present a diagnostic challenge in patients with chronic liver disease and may lead to liver resections or listing for liver transplantation.

## Conclusions

HART should be considered in the differential diagnosis of HCC in patients with chronic liver disease, especially in patients with normal alpha-fetoprotein and lesions involving hepatic segment VII.

## References

[1] Anderson JR, Ross AH (1980). Ectopic adrenal tissue in adults. Postgrad Med J.

[2] Enjoji M, Sanada K, Seki R, Ito T, Maeda M (2017). Adrenal rest tumor of the liver preoperatively diagnosed as hepatocellular carcinoma. Case Rep Surg.

[3] Sugiyama T, Tajiri T, Hiraiwa S (2015). Hepatic adrenal rest tumor: diagnostic pitfall and proposed algorithms to prevent misdiagnosis as lipid-rich hepatocellular carcinoma. Pathol Int.

[4] Wallace EZ, Leonidas JR, Stanek AE, Avramides A (1981). Endocrine studies in a patient with functioning adrenal rest tumor of the liver. Am J Med.

[5] Contreras P, Altieri E, Liberman C (1985). Adrenal rest tumor of the liver causing Cushing's syndrome: treatment with ketoconazole preceding an apparent surgical cure. J Clin Endocrinol Metab.

[6] Hyams VJ, Thomas DF, Sarkisian SS (1960). Adrenal rest tumor of the liver. Am J Surg.

[7] Arai K, Muro H, Suzuki M, Oba N, Ito K, Sasano H (2000). Adrenal rest tumor of the liver: a case report with immunohistochemical investigation of steroidogenesis. Pathol Int.

[8] Tajima T, Funakoshi A, Ikeda Y (2001). Nonfunctioning adrenal rest tumor of the liver: radiologic appearance. J Comput Assist Tomogr.

[9] Baba Y, Beppu T, Imai K, Masuda T, Iyama K, Sasano H, Baba H (2008). A case of adrenal rest tumor of the liver: radiological imaging and immunohistochemical study of steroidogenic enzymes. Hepatol Res.

[10] Shin YM (2010). Hepatic adrenal rest tumor mimicking hepatocellular carcinoma. Korean J Hepatol.

[11] Dalla Valle R, Montali F, Manuguerra R, Bresciani P (2014). Adrenal rest tumour of the liver. Dig Liver Dis.

[12] Soo KL, Azhar R, Ooi LL (2014). Hepatic adrenal rest tumour (HART): a case report. Ann Acad Med Singapore.

